# Parasitoid wasp usurps its host to guard its pupa against hyperparasitoids and induces rapid behavioral changes in the parasitized host

**DOI:** 10.1371/journal.pone.0178108

**Published:** 2017-06-21

**Authors:** Prabitha Mohan, Palatty Allesh Sinu

**Affiliations:** 1 Department of Animal Science, Central University of Kerala, Kasargod, Kerala, India; 2 Department of Ecology and Evolutionary Biology, The University of Arizona, Tucson, Arizona, United States of America; Universidade Federal de Vicosa, BRAZIL

## Abstract

Some parasites have an ability to fabricate the behavior of their host and impel the host to guard parasites' offspring, which is popularly called as bodyguard manipulation. *Psalis pennatula* larva parasitized by a braconid parasitoid wasp *Microplitis pennatula* exhibits some behavioral changes including the guarding of the parasitoid pupa from its natural enemies. We hypothesized that these behavioral change exhibited by the parasitized host larva are induced by the parasitoid and can be considered as an example of bodyguard manipulation. Even though hyperparasitoids are the more specialized natural enemy of parasitoids than predators, very few studies tested the success of guarding parasitoid pupa against hyperparasitoids. This study analyzed the success of guarding behavior of the parasitized host against hyperparasitoids. The onsets of parasite-induced phenotypic alterations (PIPAs) in the parasitized host were inspected to analyze whether these behavioral changes in the host larva manifests gradually or abruptly. The study concludes that parasitized host larva defends the parasitoid pupa from hyperparasitoids and the PIPAs in the parasitized host develops abruptly only after the egression of parasitoid prepupa.

## Introduction

Many parasites modulate the behavior and physiology of hosts for their survival and successful transmission [1]. This type of manipulation is otherwise known as the extended phenotype, where the gene of one organism has phenotypic effects in another organism [2]. Broadly, reported manipulations are of four types: (i) parasitism that lead the intermediate infected hosts to become more vulnerable to predation by its definitive hosts; e.g.: *Toxoplama gondii* infected rats become allured towards odor of cats despite its innate aversion [3]; (ii) parasites guide their hosts to atypical habitats which are suitable for the effective transmission of parasites’ propagules; e.g. ‘Suicide’ of *Paragordius tricuspidatus*-parasitized *Nemobius sylvestris* in water bodies [4]; (iii) some parasites modulates feeding behavior of hosts, which act as their vectors; e.g. Plasmodium escalates feeding frequency of mosquitoes which in turn increases the transmission rate of parasite itself [5]; and (iv) parasites manipulate the behavior of host in such a way that they guard the developing parasite against natural enemies, which is otherwise called as bodyguard manipulation; e.g. parasitized *Pieris brassicae* spins a web over the cocoon of its parasitoid *Cotesia glomerata*, which provides protection to parasitoid from its natural enemies [6].

Parasitoids use different strategies to protect their vulnerable pupal stage from natural enemies like predators and hyperparasitoids. *Aphidius nigripes*, a parasitoid maneuvers its aphid host from their host plants to a concealed place to mummify [7]. Bodyguarding behavior is one such strategy induced by the parasitoids, precisely koinobionts, which feeds only on haemolymph and fat body of the host tissue [8]. Manipulated host responds aggressively and shakes off the approaching predators and hyperparasitoids [6, 9–11]. Interestingly, most of the previous studies reported the success stories in the host-parasitoid-predator interaction while only a few assessed it with hyperparasitoids [9]

Manipulative parasites can induce several phenotypic alterations [PIPAs] in the parasitized host [12], which vary in its magnitude and diversity. Parasitoids can also induce some phenotypic alterations in its host like increased defensive response, stoppage of feeding and walking. Few studies reported that these changes were observed only after the egression of parasitoid [10, 13], but none had experimentally studied whether these changes were accumulating gradually in the parasitized host or abruptly developed.

**Fig 1:**
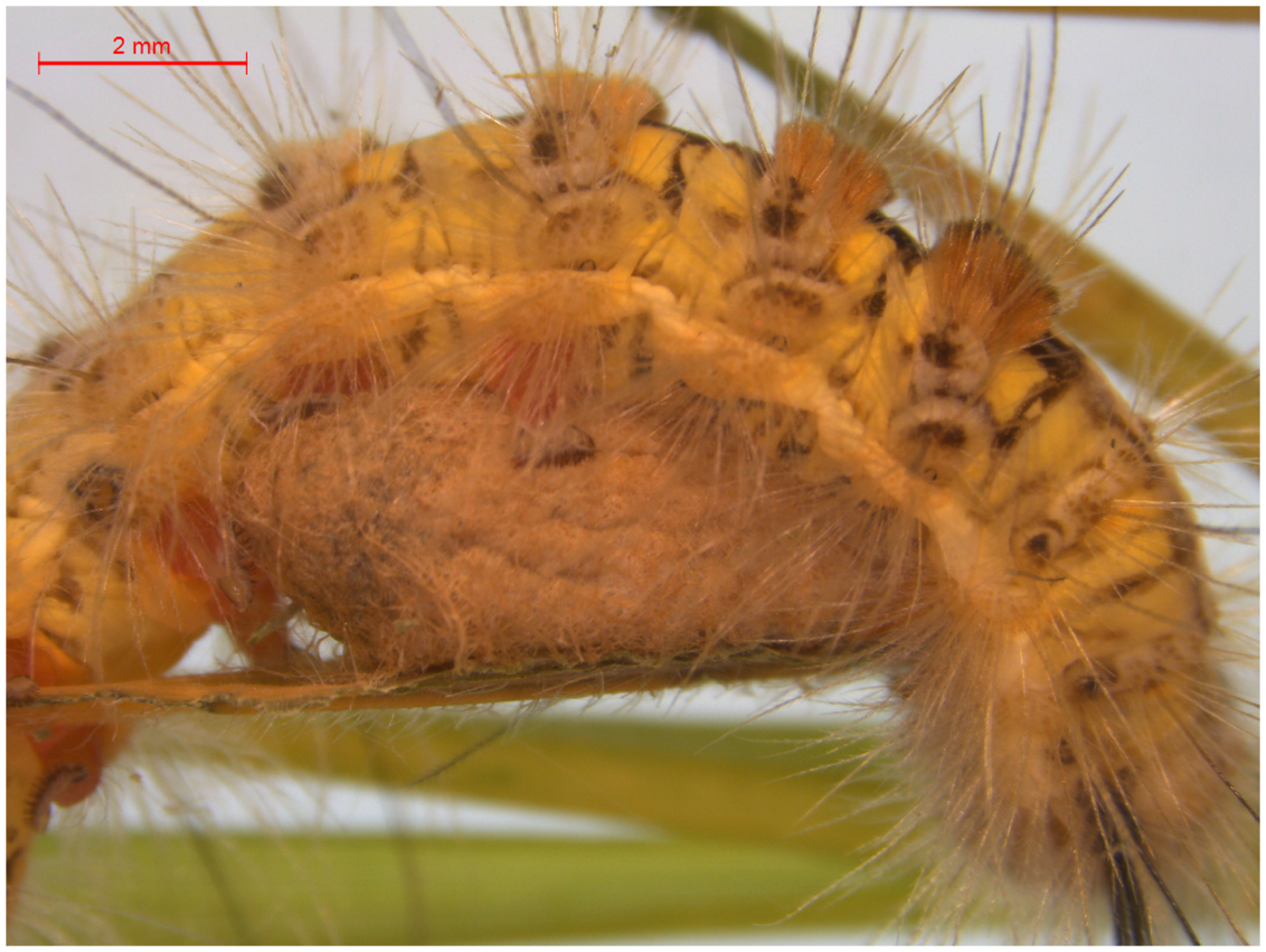
Parasitoid cocoon of *Microplitis pennatula* (Braconidae) is finding a refuge under the host larva of *Psalis pennatula* (Erebidae).

*Psalis pennatula* Fabricius (Lepidoptera: Erebidae) parasitized by its parasitoid, *Microplitis pennatula* Ranjith & Rajesh (Hymenoptera: Braconidae) exhibited some behavioral changes. *M*. *pennatula* prepupa emerges from a still-living host larva of *P*. *pennatula* and pupates under it, near the abdominal prolegs (Fig 1). After parasitoid egression, the host larva remains alive for 80 h (in laboratory conditions) and responds aggressively to any external disturbances by its vigorous head swings. We hypothesized that this behavioral change in the host larva is induced by the parasitoid to guard its offspring. Although hyperparasitoid insects are relatively specialized natural enemies of parasitoid larvae or pupae than the insect predators, very few studies had examined how parasitized host combat and guard parasitoid’s offspring against hyperparasitoids; in this study, we report such a case. We also inquired about other parasite-induced phenotypic alterations (PIPAs) in the parasitized host and examined whether those were building up gradually in the host larva after parasitization and materialize after the parasitoid prepupal egression.

## Materials and methods

### Rearing protocol

*Psalis pennatula*, *Microplitis pennatula*, and *Brachymeria* sp. were collected from the paddy fields of Regional Agriculture Research Station, Pattambi (10° 48' N, 76° 11' E), Kerala, South India. Caterpillars collected were reared on leaf blades of potted paddy plants (variety: ‘Uma’). Adult male and female moths emerged from pupae were transferred to a mating chamber provided with paper cards for laying eggs. The neonate caterpillars of *P*. *pennatula* soon after its emergence (6–7 days) were transferred again to the cages with their natural diet. The field-collected *M*. *pennatula* pupae were kept in falcon tubes until the adult wasps emerged out. Adult male and female *M*. *pennatula* wasps were transferred to test tubes for mating. The mated female wasps were provided with the third instar host larva for parasitization. After visual confirmation of parasitization, parasitized larva was replaced by unparasitized larvae. Parasitized larvae were transferred to separate cages and were reared on the natural diet. The larva of *M*. *pennatula* completes its development inside the host and egresses within 11–14 days (under laboratory conditions) and pupates. Hyperparasitoids collected from the field were allowed to mate in the glass test tubes and the female wasps were provided with <48 hours old *M*. *pennatula* pupae for hyperparasitization. Parasitoids and hyperparasitoids were provided with diluted honey 10% (w/v) on thick plastic strips and moths with 10% (w/v) honey on moistened filter paper. All insects were reared under ambient temperature and light conditions and the culture were constantly supplemented with field collected samples. Host larvae emerged from a single batch of eggs laid by a single female were used to study the growth and behavior of the parasitized and unparasitized host caterpillars in different experimental conditions.

### Hyperparasitization experiments to test bodyguard hypothesis

Female *Brachymeria* sp. was introduced to the following two experimental conditions (a) parasitoid pupa mounted by an attending host larva (N = 20), (b) parasitoid pupa alone (the host larva was removed before the hyperparasitoid was introduced) (N = 10) [9]. The success of guarding behavior of host larva was assessed by analyzing the number of pupae hyperparasitized within a given time. The time taken by the hyperparasitoids to get access to the parasitoid pupa in aforesaid conditions was also compared. All the *M*. *pennatula* pupae exposed to the hyperparasitoids were <48 hours old after their egression, as that is the most susceptible period for hyperparasitization (personal observation). Hyperparasitization was inspected for 300 seconds based on the preliminary observation that *Brachymeria* sp. could detect the parasitoid pupa in a test tube within that time. The success of hyperparasitization and the time taken for hyperparasitization were tested using the Fisher’s exact test and Wilcoxon rank sum test, respectively.

### Behavior of manipulated host larva

The behavior of two types of parasitized larvae [before (BE) and after (AE) the egression of parasitoid prepupa] was compared with that of unparasitized larvae (UP) of same cohort and age to examine the effect of parasitization. The instant of change in routine activities such as feeding and walking and the appearance of defensive response in the aforesaid larvae were analyzed using generalized linear models. The host larval type was fitted as the fixed effect in different models; wherever the count was the type of the response variable, we fitted Poisson distribution as the error type, and wherever a continuous measure was the type of response variable, we used the default Gaussian distribution as the error type in the model.

To examine the tendency for deterioration in feeding and walking activities, host larvae of the following age groups: (i) 5 days after parasitization (the larvae at that time were at its 3^rd^ instar) (ii) 10 days after parasitization (then larvae were usually in its 4^th^ instar) were compared with the control group of unparasitized host larvae of the same cohort and age. The rate of feeding was compared using Mann-Whitney U-test and the rate of walking was compared using student t-test. All the analyses were performed in R 3.1.2 [14].

#### Feeding and walking tests

Host larvae starved for 12 hours were introduced to test tubes containing pre-weighed natural diet and allowed to feed for 4 hours. The difference in the weight of diet after feeding was calculated. In control test tubes the pre-weighed diet was kept for 4 hours without larvae to account for the natural variation in the weight change in diet due to moisture loss [15]. The amount of food consumed by a given larva was calculated using the following equations.

Amount of food consumed (mg)=weight change due to feeding (mg) − weight change due to moisture loss in control leaves (mg)

Rate of feeding (mg/hour)=amount of food consumed (mg)/time of test (i.e. 4 hours)

Walking rate in host larvae was examined using a customised protocol of Grosman (2008). Briefly, the rate of locomotion was calculated by recording the time taken by the host larva to cover 50 cm distance on a cotton packing string tied two sides on wooden stands. The starting and finishing points were marked using coloured tags. The head of the larva was kept behind the starting point and the time taken to complete the finishing point was noted. Those larvae that halted in between and that walked in opposite direction were excluded from the experiment.

#### Origin of defensive response

The provenance of defensive response in the host larvae was tested by analysing the response of three categories of host larvae (BE, AE and UP) to a simulated attack. The larvae tested were touched consecutively with the fine bristles of a paint brush (#2) [9] and recorded the number of swings made by it. The larvae that vigorously swung or thrashed their body to the probing brush hairs were listed as defensive larvae and those not twitched, but, normally walked were listed as non-responsive larvae.

### Field permit

Field permit for doing sample collection in RARS, Pattambi was granted by Dr. K. Karthikeyan, Associate Professor, Regional Agricultural Research Station, Pattambi. The field study does not involve any endangered species.

## Results

**Fig 2:**
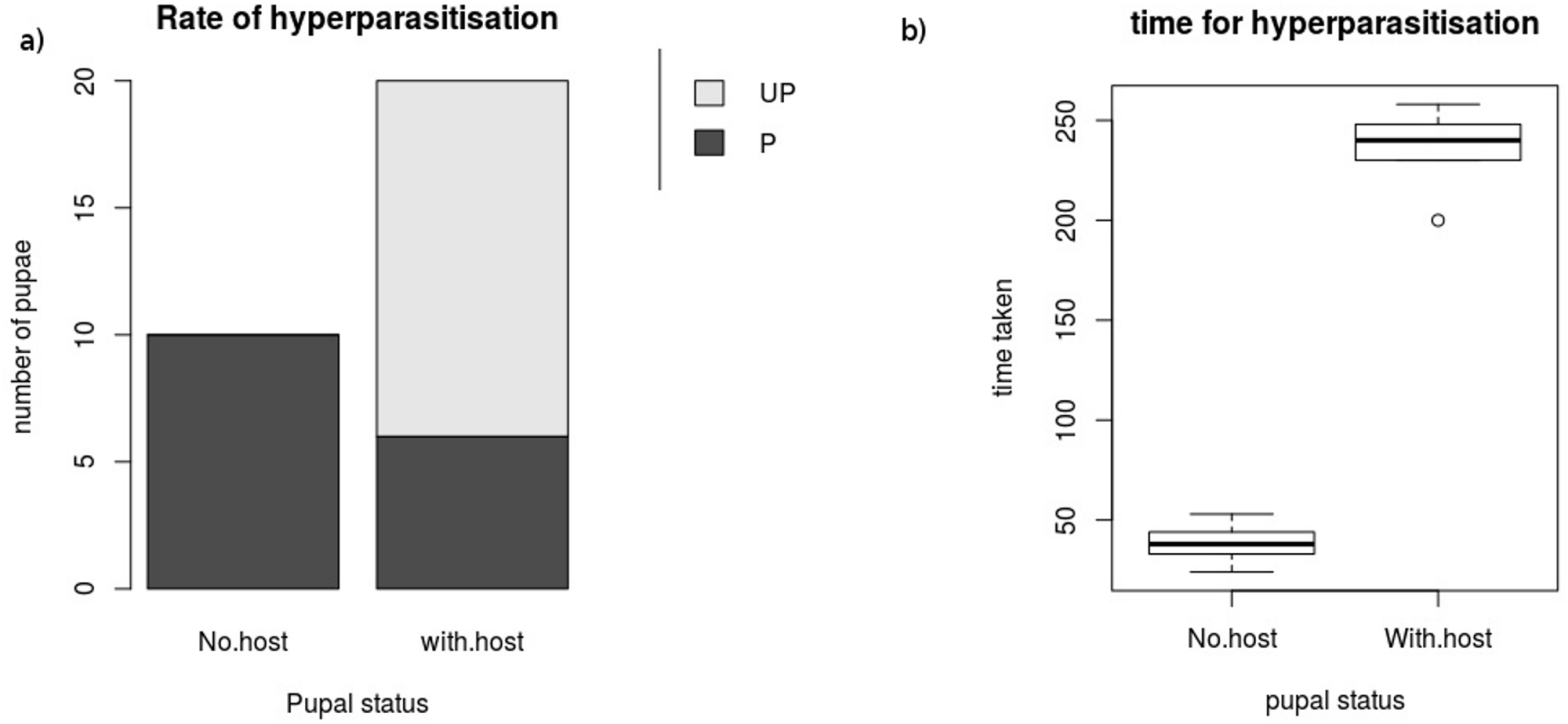
The (a) rate of hyperparasitization and (b) the time taken for hyperaparsitization by *Brachymeria* sp. to hyperparasitze the pupa of *Microplitis pennatula* with and without host larva.

Brachymeria sp. readily hyperparasitized all the unguarded parasitoid pupae while it could only hyperparasitize 6 guarded parasitoid pupae (Fisher’s exact test: p-value = 0.0002). The time took for hyperparasitizing guarded pupae (236± 8.1 sec) were also significantly higher than the time taken for unguarded pupae (38.1± 2.8 sec) (Wilcoxon rank sum test: W = 60, p = 0.001) (Fig 2; S1 Table).

**Fig 3:**
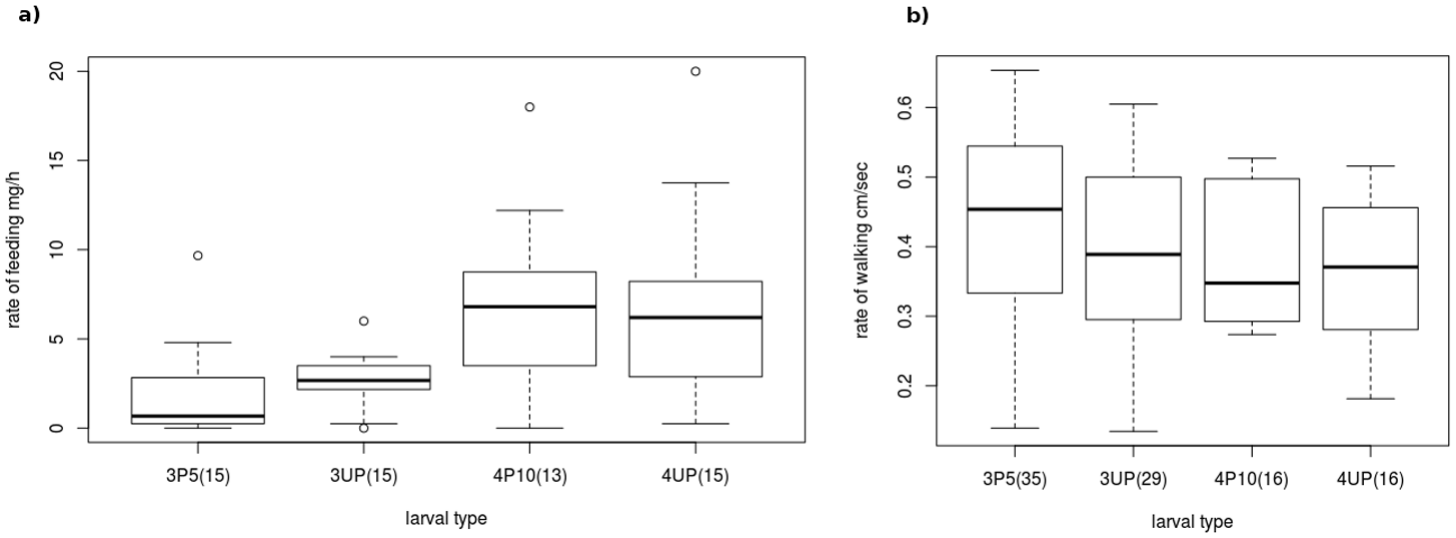
Rate of feeding **(a)** and walking **(b)** by unparasitized (UP) and parasitized (P) host larvae. The numbers preceding and succeeding the host larva type denote the instar stage and days after parasitization, respectively. The numbers in parentheses are the sample sizes. The rates of feeding (3^rd^ instar: Mann-Whitney U-test, U = 81.5, p = 0.198; 4^th^ instar: Mann-Whitney U-test, U = 90, p = 0.539) and walking (3^rd^ instar: t-test, t_61.81_ = 0.66, p = 0.51; 4^th^ instar: t-test, t_29.811_ = 0.30, p = 0.76) were not different between parasitized and unparasitized host larvae of any given age.

**Fig 4:**
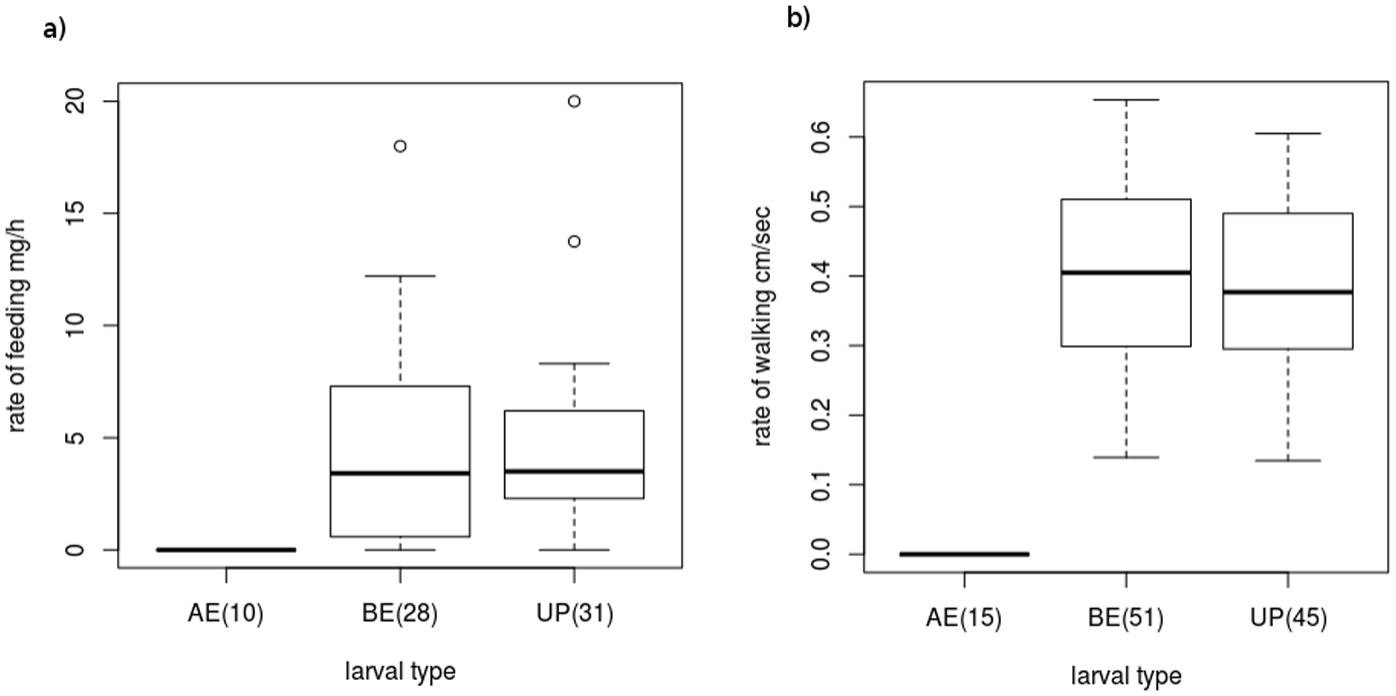
Rate of feeding **(a)** and walking **(b)** of unparasitized (UP) and parasitized host larva of *P*. *pennatula* before (BE) and after (AE) the egression of the parasitoid. The numbers in the parentheses denote the number of samples.

The feeding and walking activities of the parasitized host larvae of 3^rd^ and 4^th^ instar were absolutely normal as that of unparasitized host larvae of the same age (Fig 3; S2 Table). The parasitized host larva walked normally like an unparasitised host larva (GLM: F_1,94_ = 0.735, p = 0.39) until the parasitoid prepupa egressed from the body of the host larva, since when the host larva stopped walking (GLM: F_2,108_ = 76.49, p<0.0001). Similarly, the parasitized host larva fed normally like an unparasitised host larva (GLM: F_1,57_ = 0.05, p = 0.83) until the parasitoid prepupa egressed from the body of the host (GLM: F_2,66_ = 5.39, p = 0.007). (Fig 4; S3 Table)

**Fig 5:**
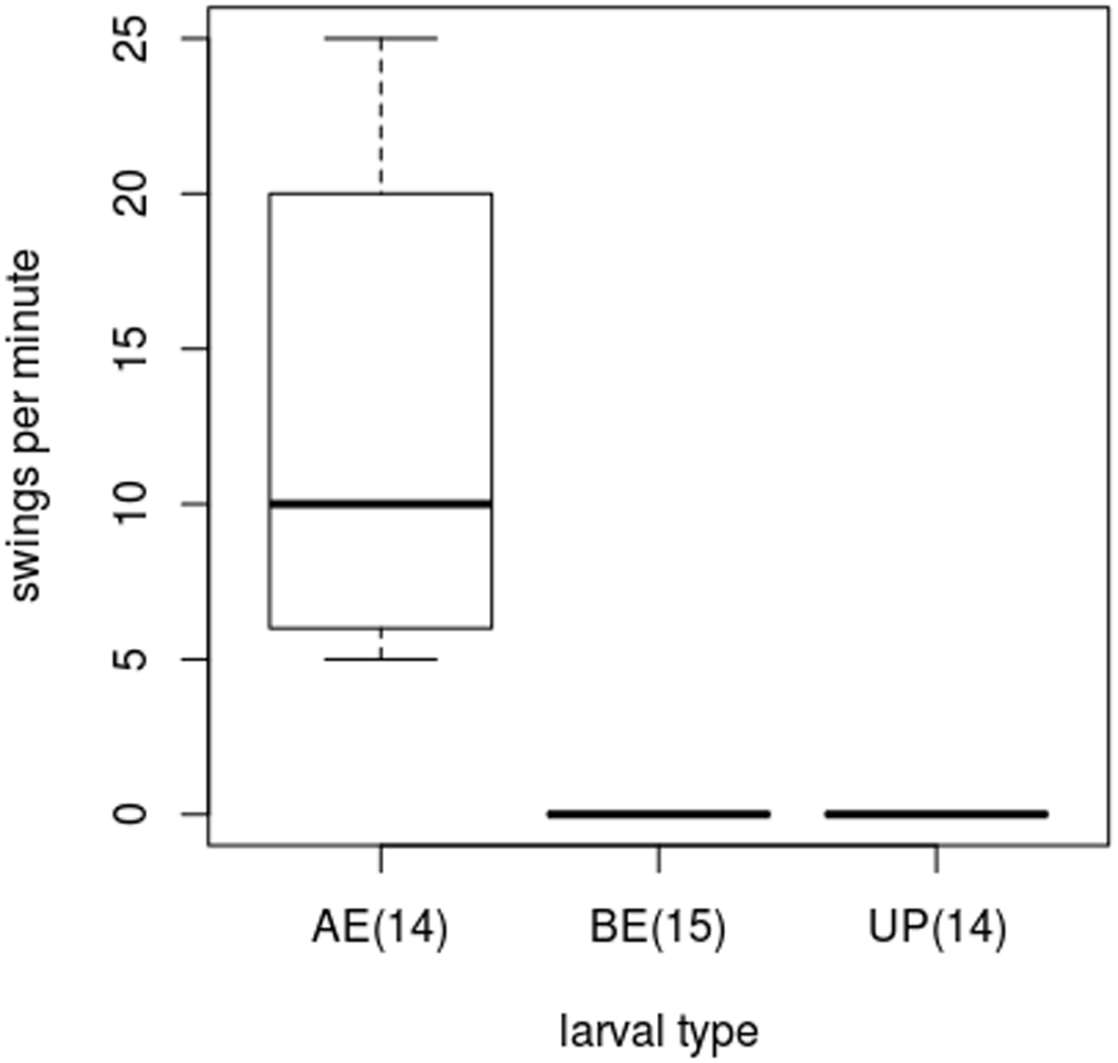
The host larvae’s defensive response as measured by the number of swings was significantly higher after the emergence of the parasitoid prepupa.

Similarly, the unparasitized host larvae (UP) and the parasitized host larvae before the egression of parasitoid prepupa (BE) did not display any remarkable response to the simulated attack by probing brush hairs. However, all the parasitized host larvae after the egression of the parasitoid prepupa (AE) became defensive and displayed twitching response to the probing brush hairs (GLM: F_2,40_ = 132.06, p<0.0001) (Fig 5; S4 Table).

## Discussion

The present study documents a novel case of bodyguard manipulation in a lepidopteran rice pest, *P*. *pennatula* by a braconid parasitoid, *M*. *pennatula*. We investigated whether the manipulated host larvae could guard the pupae of *M*. *pennatula* during its most vulnerable period from its hyperparasitoids. In support of our hypothesis, results show that the live bodyguard host larva often fails or delays the hyperaparsitisation attempts of *Brachymeria* sp. The response of the host larva is extremely vigorous that the hyperparasitoids attempting to hyperparasitize are literally thrown away from the pupa (S1 Video).

The feeding and walking activities of the host larvae were not at all affected by the parasitization during the course of development of the parasitoid larva inside the host body, rather, it dramatically came to a halt upon the egression of the parasitoid prepupa. Our laboratory experiments also show that the defensive character in the host larvae developed only after the egression of the parasitoid. Thus, we establish that behavioral manipulation is not a gradually developing process within the host larva, but an abruptly developed change upon the egression of parasitoid prepupa.

The defensive response of the parasitized host, time took by the hyperparasitoid to approach a guarded pupa and fate of an unguarded pupa in laboratory conditions indicates that behavioral manipulations definitely make the guarded parasitoid pupae a less attractive target to the hyperparasitoids and clearly increase the fitness of the parasitoid. So it could be considered as a case of bodyguard manipulation like similar reports on other parasitic wasps [6, 9–11, 13]. Interestingly, another parasitoid of *P*. *pennatula*, Charops sp. (Ichneumonidae) which was also collected in the present study did not induce any kind of manipulative behavior in the host larvae. Unlike *M*. *pennatula*, Charops sp. kills the host upon its egression (personal observation) and pupates in a cocoon suspended from the leaf blade. This suggests that these behavioral modifications exhibited by the *P*. *pennatula* due to the parasitization of *M*. *pennatula* are specific to the parasitoid.

Since these remarkable manipulated behaviors are expressed only by the post-parasitoid-egressed host larvae, it is clear that these changes are not directly induced by the parasitoid but likely to be induced by another agent [16]. Earlier studies report that a symbiotic bracovirus seen in the calyx fluid of some braconid wasps prompts suppression of the immune response in the host and facilitates the growth of parasitoid inside the host [17]. The presence of a symbiont bracovirus was earlier reported in a related *Microplitis* species, *M*. *croceipes* which are also capable of manipulating their host behavior [18]. Recently, it was reported that a symbiont RNA virus in a braconid wasp *Dinocampus coccinellae* might be a true manipulator of the behavior of its ladybird beetle host [16, 19]. Future investigations might be required to unravel the complete mechanism behind the manipulative behavior in *P*. *pennatula*.

## Supporting information

S1 Video Defensive response of parasitized host larva towards approaching hyperparasitoids. (MP4)

S1 Table Time taken for hyperparasitization in successful hyperparasitization events. (PDF)

S2 Table Rate of feeding (a) and walking (b) by of larvae of different ages and stages. (PDF)

S3 Table Rate of feeding (a) and walking (b) of unparasitized (UP) and parasitized host larva before (BE) and after (AE) the egression of the parasitoid. (PDF)

S4 Table Defensive response of host larvae of different stages. (PDF)
