# Supplementary material for: Parasitoid wasp usurps its host to guard its pupa against hyperparasitoids and induces rapid behavioral changes in the parasitized host
Source: PLoS One. 2017 Jun 21;12(6):e0178108. doi: 10.1371/journal.pone.0178108 (PMC5479522; doi:10.1371/journal.pone.0178108)
Supplement: S1 Table — (PDF) [file pone.0178108.s002.pdf]

S1 Table

Data used for Fig 2

Legend: Time taken for hyperparasitization in successful hyperparasitization events (UP: unparasitized)

With.host (n=20)	No.host (n=10)
200	38
230	38
240	24
240	40
248	27
258	36
UP	44
UP	53
UP	33
UP	48
UP	
UP	
UP	
UP	
UP	
UP	
UP	
UP	
UP	
UP	
