# Supplementary material for: Parasitoid wasp usurps its host to guard its pupa against hyperparasitoids and induces rapid behavioral changes in the parasitized host
Source: PLoS One. 2017 Jun 21;12(6):e0178108. doi: 10.1371/journal.pone.0178108 (PMC5479522; doi:10.1371/journal.pone.0178108)
Supplement: S2 Table — (PDF) [file pone.0178108.s003.pdf]

S2 Table

Data used for Fig 3

a) **Rate of feeding of larvae of different ages and stages (mg/h)**

Legend: 3P5 – 3rd instar larvae, 5 days after paratisation; 3 UP – 3rd instar unparasitized larva; 4UP – 4th instar unparasitized larva; 4P10 – 4th instar, 10 days after parasitisation

3P5	3UP	4UP	4P10
0	2.33	0.25	0
0.67	0	3.5	3.5
3.33	2.67	7.25	18
2.33	2.5	6	7.8
9.67	6	8.25	3.5
4	3.5	1.75	8.75
0.25	0.25	2.25	10
0.25	2	20	12.2
0	4	13.75	6.75
0.5	3.5	5.85	3.25
1	2.8	8.3	6.8
4.8	2.8	6.2	8.75
0.25	0.25	7.25	3.75
0.67	2.33	6.2	
2.33	3.5	8.2	

b) Rate of walking of larvae of different ages and stages (cm/sec)

3P5	3UP	4P10	4UP
0.505	0.583	0.505	0.505
0.343	0.454	0.510	0.353
0.208	0.318	0.327	0.495
0.139	0.490	0.495	0.360
0.145	0.295	0.527	0.377
0.262	0.135	0.383	0.280
0.345	0.500	0.405	0.371
0.266	0.605	0.304	0.516
0.389	0.415	0.290	0.331
0.278	0.380	0.302	0.340
0.476	0.521	0.285	0.282
0.333	0.226	0.285	0.322
0.419	0.383	0.500	0.280
0.527	0.275	0.368	0.405
0.544	0.251	0.274	0.331
0.490	0.389	0.295	0.265
0.598	0.377		
0.480	0.333		
0.415	0.430		
0.570	0.521		
0.510	0.343		
0.454	0.209		
0.380	0.293		
0.583	0.538		
0.598	0.576		
0.653	0.471		
0.583	0.249		
0.551	0.490		
0.495	0.583		
0.544			
0.371			
0.645			
0.180			
0.333			
0.224			
