# Supplementary material for: Parasitoid wasp usurps its host to guard its pupa against hyperparasitoids and induces rapid behavioral changes in the parasitized host
Source: PLoS One. 2017 Jun 21;12(6):e0178108. doi: 10.1371/journal.pone.0178108 (PMC5479522; doi:10.1371/journal.pone.0178108)
Supplement: S3 Table — (PDF) [file pone.0178108.s004.pdf]

S3 Table

**Data used for Fig 4**

a) **Rate of feeding of host larva of different stages (mg/h)**
Legend (BE – Before egression of parasitoid prepupa; UP – Unparasitized host larva; AE – After egression of parasitoid prepupa)

| UP | BE | AE |
|---|---|---|
| 2.33 | 0 | 0 |
| 0 | 0.67 | 0 |
| 2.67 | 3.33 | 0 |
| 2.5 | 2.33 | 0 |
| 6 | 9.67 | 0 |
| 3.5 | 4 | 0 |
| 0.25 | 0.25 | 0 |
| 2 | 0.25 | 0 |
| 4 | 0 | 0 |
| 3.5 | 0.5 | 0 |
| 2.8 | 1 | |
| 2.8 | 4.8 | |
| 0.25 | 0.25 | |
| 2.33 | 0.67 | |
| 3.5 | 2.33 | |
| 0.25 | 0 | |
| 3.5 | 3.5 | |
| 7.25 | 18 | |
| 6 | 7.8 | |
| 8.25 | 3.5 | |
| 1.75 | 8.75 | |
| 2.25 | 10 | |
| 20 | 12.2 | |
| 13.75 | 6.75 | |
| 5.85 | 3.25 | |
| 8.3 | 6.8 | |
| 6.2 | 8.75 | |
| 7.25 | 3.75 | |
| 6.2 | | |
| 8.2 | | |
| 0.25 | | |

**b) Rate of walking of host larva of different stages (cm/sec)**

| UP | BE | AE |
|---|---|---|
| 0.583 | 0.505 | 0 |
| 0.454 | 0.343 | 0 |
| 0.318 | 0.208 | 0 |
| 0.490 | 0.139 | 0 |
| 0.295 | 0.145 | 0 |
| 0.135 | 0.262 | 0 |
| 0.500 | 0.345 | 0 |
| 0.605 | 0.266 | 0 |
| 0.415 | 0.389 | 0 |
| 0.380 | 0.278 | 0 |
| 0.521 | 0.476 | 0 |
| 0.226 | 0.333 | 0 |
| 0.383 | 0.419 | 0 |
| 0.275 | 0.527 | 0 |
| 0.251 | 0.544 | 0 |
| 0.389 | 0.490 | |
| 0.377 | 0.598 | |
| 0.333 | 0.480 | |
| 0.430 | 0.415 | |
| 0.521 | 0.570 | |
| 0.343 | 0.510 | |
| 0.209 | 0.454 | |
| 0.293 | 0.380 | |
| 0.538 | 0.583 | |
| 0.576 | 0.598 | |
| 0.471 | 0.653 | |
| 0.249 | 0.583 | |
| 0.490 | 0.551 | |
| 0.583 | 0.495 | |
| 0.505 | 0.544 | |
| 0.353 | 0.371 | |
| 0.495 | 0.645 | |
| 0.360 | 0.180 | |
| 0.377 | 0.333 | |
| 0.280 | 0.224 | |
| 0.371 | 0.505 | |
| 0.516 | 0.510 | |
| 0.331 | 0.327 | |
| 0.340 | 0.495 | |
| 0.282 | 0.527 | |
| 0.322 | 0.383 | |
| 0.280 | 0.405 | |

| | | |
|---|---|---|
| 0.405 | 0.304 | |
| 0.331 | 0.290 | |
| 0.265 | 0.302 | |
| | 0.285 | |
| | 0.285 | |
| | 0.500 | |
| | 0.368 | |
| | 0.274 | |
| | 0.295 | |
