# Supplementary material for: Parasitoid wasp usurps its host to guard its pupa against hyperparasitoids and induces rapid behavioral changes in the parasitized host
Source: PLoS One. 2017 Jun 21;12(6):e0178108. doi: 10.1371/journal.pone.0178108 (PMC5479522; doi:10.1371/journal.pone.0178108)
Supplement: S4 Table — (PDF) [file pone.0178108.s005.pdf]

S4 Table

Data used for Fig 5 (Defensive response of host larvae of different stages (number of head swings/min)

Legend as for Table 4

UP	BE	AE
0	0	8
0	0	12
0	0	6
0	0	23
0	0	5
0	0	25
0	0	20
0	0	15
0	0	20
0	0	5
0	0	6
0	0	5
0	0	19
0	0	6
	0	
